# Draft Genome Sequences of 24 Microbial Strains Assembled from Direct Sequencing from 4 Stool Samples

**DOI:** 10.1128/genomeA.00526-15

**Published:** 2015-05-28

**Authors:** Patricio Jeraldo, Álvaro Hernández, Bryan A. White, Daniel O’Brien, David Ahlquist, Lisa Boardman, Nicholas Chia

**Affiliations:** a Department of Surgery, Mayo Clinic, Rochester, Minnesota, USA; b Center for Individualized Medicine, Mayo Clinic, Rochester, Minnesota, USA; c Carl R. Woese Institute for Genomic Biology, University of Illinois at Urbana-Champaign, Urbana, Illinois, USA; d Roy J. Carver Biotechnology Center, University of Illinois at Urbana-Champaign, Urbana, Illinois, USA; e Department of Animal Sciences, University of Illinois at Urbana-Champaign, Urbana, Illinois, USA; f Department of Health Sciences Research, Mayo Clinic, Rochester, Minnestota, USA; g Division of Gastroenterology and Hepatology, Mayo Clinic, Rochester, Minnesota, USA

## Abstract

The ability to assemble genomes from metagenomic sequencing avoids the need for culture and any associated culture biases. We assembled 24 essentially complete draft genomes from metagenomic pair-end and size-selected mate pair sequencing from 4 stool samples, 2 from subjects diagnosed with colorectal cancer and 2 from healthy controls.

## GENOME ANNOUNCEMENT

Colorectal cancer is common, with 136,830 new cancers annually, and lethal, with 51,310 cancer deaths each year (1), and has plausible connections to several microbial agents. There are multiple ongoing efforts to identify microbes that cause cancer and find ways to quantify that risk by using a combination of modern computational and sequencing techniques in the hopes of eventually attenuating that risk by manipulating the gut microbiome using antibiotics, probiotics, or prebiotics. This would radically change the way colon cancer is treated.

Subjects were consented under IRB number 10-006009, which was reviewed and approved by the Mayo Clinic Institutional Review Board. Written consent was provided by all subjects. Genomic DNA was extracted from 4 stool samples collected from subjects prior to colonoscopy. Two were from subjects who were diagnosed with colon cancer, and two were from subjects with a negative colonoscopy. Paired-end and Nextera mate pair DNA libraries from each sample were prepared with target fragment lengths of 500 bp for the paired-end library, and 4 to 6 kb, 8 to 10 kb and 15 to 20 kb for the mate pair libraries. A total of 9,195,789,522 reads were produced on Illumina HiSeq 2500 runs for 300 cycles.

From this, for the reference-based binning and assembly, we iteratively mapped the reads onto the MetaHIT metagenomic species references (2) using BWA 0.7.10 (3), assembled each bin using SPAdes 3.5.0 (4), and removed extraneous contigs using BioBloom Tools 2.0.6 (5). For the *de novo* binning and assembly, we assembled the whole metagenome using Ray 2.3.1 (6), for a total of 2.1 Gbp assembled into 1,748,414 contigs, with an *N*_50_ of 40,874 bp. Contigs were binned using differential coverage, taxonomy, G+C content, and tetranucleotide frequency (7), unassembled reads were mapped onto these bins using BWA 0.7.10, and each bin was reassembled using SPAdes 3.5.0.

From these 353 genomic bins, we filtered for essentially complete, high-fidelity genomes satisfying all the following criteria. Genomes must have at least 100 of a set of essential genes (7), have single copies of a set of 24 single-copy genes (8) allowing for two extra copies, having at least cone copy of the 16S rRNA gene longer than 1,300 bp, and having at most 12 copies of complete or partial 16S rRNA genes. Genome completeness was estimated using CheckM 0.9.7 (9) This resulted in 24 essentially complete genomes, listed in Table 1.

**TABLE 1:** List of accession numbers for genomes submitted under this announcement

Nomenclature	BioSample	Accession no.	Total no. of bases	*N*_50_	Estimated completeness (%)
*Ruminococcus* sp. A254.MGS-108	SAMN03449993	LAQZ00000000	1,942,857	61,188	97.32
*Clostridium* sp. A254.MGS-251	SAMN03449994	LARA00000000	1,983,932	78,179	98.66
*Burkholderia* sp. K4410.MGS-135	SAMN03449996	LARC00000000	2,654,415	49,325	100
*Clostridium* sp. K4410.MGS-306	SAMN03449997	LARD00000000	2,054,369	124,392	89.74
*Odoribacter* sp. N15.MGS-14	SAMN03449998	LARE00000000	3,441,564	42386	98.83
*Ruminococcus* sp. N15.MGS-57	SAMN03450004	LARF00000000	2,359,762	45,122	97.95
*Alistipes* sp. N15.MGS-157	SAMN03449999	LARG00000000	2,171,493	171,798	99.52
*Carnobacterium* sp. N15.MGS-207	SAMN03450000	LARH00000000	2,235,056	71,777	99.98
*Carnobacterium* sp. N15.MGS-251	SAMN03450001	LARI00000000	1,977,841	102,358	98.66
*Lactobacillus* sp. N15.MGS-260	SAMN03450002	LARJ00000000	1,820,858	58,043	94.68
*Heliobacterium* sp. N15.MGS-306	SAMN03450003	LARK00000000	2,061,532	121,894	89.74
*Odoribacter* sp. N54.MGS-14	SAMN03450006	LARL00000000	3,456,951	38,252	98.57
*Bacteroides* sp. N54.MGS-20	SAMN03450007	LARM00000000	2,979,725	74,275	99.96
*Acinetobacter* sp. N54.MGS-139	SAMN03450005	LARN00000000	2,171,676	41,165	100
*Lactobacillus* sp. N54.MGS-719	SAMN03450008	LARO00000000	1,821,790	36,466	98.72
*Akkermansia* sp. UNK.MGS-1	SAMN03456685	LBCB00000000	2,770,805	190,924	92.12
*Clostridium* sp. UNK.MGS-6	SAMN03456689	LBCC00000000	2,411,497	19,226	87.94
*Odoribacter* sp. UNK.MGS-12	SAMN03456680	LBCD00000000	5,872,338	91,532	95.72
*Bacteroides* sp. UNK.MGS-14	SAMN03456682	LBCF00000000	5,132,637	250,314	98.75
*Roseburia* sp. UNK.MGS-15	SAMN03456683	LBCG00000000	4,890,836	278,604	93.1
*Rhodospirillum* sp. UNK.MGS-17	SAMN03456684	LBCH00000000	1,897,358	219,441	94.68
*Eubacterium* sp. UNK.MGS-25	SAMN03456686	LBCI00000000	4,343,513	459,237	97.96
*Eubacterium* sp. UNK.MGS-26	SAMN03456687	LBCJ00000000	4,451,140	3,090,758	97.96
*Ruminococcus* sp. UNK.MGS-30	SAMN03456688	LBCK00000000	2,959,500	615,138	87.25

### Nucleotide sequence accession numbers.

These complete genome sequences have been deposited at DDBJ/EMBL/GenBank under the accession numbers provided in Table 1.
